# *In silico* PCR analysis: a comprehensive bioinformatics tool for enhancing nucleic acid amplification assays

**DOI:** 10.3389/fbinf.2024.1464197

**Published:** 2024-10-07

**Authors:** Ruslan Kalendar, Alexandr Shevtsov, Zhenis Otarbay, Aisulu Ismailova

**Affiliations:** ^1^ Helsinki Institute of Life Science (HiLIFE), University of Helsinki, Biocentre 3, Helsinki, Finland; ^2^ National Laboratory Astana, Nazarbayev University, Astana, Kazakhstan; ^3^ National Center for Biotechnology, Astana, Kazakhstan; ^4^ Astana IT University, Astana, Kazakhstan; ^5^ Department Information Systems, S. Seifullin Kazakh Agro Technical Research University, Astana, Kazakhstan

**Keywords:** *In silico* PCR, genotyping, PCR primer and probe analysis, degenerate PCR, bisulfite conversion

## Abstract

Nucleic acid amplification assays represent a pivotal category of methodologies for targeted sequence detection within contemporary biological research, boasting diverse utility in diagnostics, identification, and DNA sequencing. The foundational principles of these assays have been extrapolated to various simple and intricate nucleic acid amplification technologies. Concurrently, a burgeoning trend toward computational or virtual methodologies is exemplified by *in silico* PCR analysis. *In silico* PCR analysis is a valuable and productive adjunctive approach for ensuring primer or probe specificity across a broad spectrum of PCR applications encompassing gene discovery through homology analysis, molecular diagnostics, DNA profiling, and repeat sequence identification. The prediction of primer and probe sensitivity and specificity necessitates thorough database searches, accounting for an optimal balance of mismatch tolerance, sequence similarity, and thermal stability. This software facilitates *in silico* PCR analyses of both linear and circular DNA templates, including bisulfited treatment DNA, enabling multiple primer or probe searches within databases of varying scales alongside advanced search functionalities. This tool is suitable for processing batch files and is essential for automation when working with large amounts of data.

## Introduction

Nucleic acid amplification assays are an essential class of specific target sequence detection methods in modern biology, with diverse applications in diagnosing inherited diseases, human identification, microorganism identification, genotyping, and DNA sequencing (Khodakov et al., 2021). Currently, several thermocycling and isothermal techniques exist for nucleic acid amplification. The polymerase chain reaction (PCR) amplification method allows the production and detection of target nucleic acid sequences with high sensitivity and specificity. Methods for detecting a PCR product (amplicon) using an oligonucleotide probe capable of hybridizing to the target sequence or amplicon. Several isothermal techniques that do not rely on thermocycling to drive the amplification reaction have also been developed (Li and Macdonald, 2015). Other methods for detecting target sequences involve a probe (quantitative PCR) or microarrays that have been modified so that they can be detected during an amplification reaction (Kolpashchikov, 2019). TaqMan and Molecular Beacons assays both use a reporter and a quencher dye attached to the probe. TaqMan probes hybridize to the target sequence during amplification. The enzyme that amplifies a target sequence also degrades any hybridized probe. Conventional molecular beacons are single-stranded oligonucleotide hybridization probes that form a stem-and-loop structure. The loop contains the sequence complementary to the target nucleic acid (either DNA or RNA). The stem is formed by hybridizing the complementary sequence of the 3′-end to the 5′-end. The ends of a molecular beacon are self-complementary and are not designed to hybridize to a target sequence. A pair of different or the same oligonucleotides (primers) is required to perform a PCR reaction. Primer design is critical in all PCR methods to ensure specific and efficient target sequence amplification. The first (forward primer) binds to one strand of DNA, and the second (reverse primer) binds to the complementary strand. Assume that an appropriate distance separates the pair of sites that match the primers. In this case, the DNA fragment between these sites (known as the PCR product or amplicon) is copied by the polymerase, approximately doubling in size during each cycle. An implicit assumption is that stable hybridization of a primer to the template is a prerequisite for priming by the DNA polymerase. Therefore, the correct selection of primers is a critical step in the PCR process. The accuracy of *in silico* calculations of the interactions between primers and DNA templates is critical for predicting virtual PCR results. Isothermal techniques using DNA polymerases with strand displacement activity have emerged as a nucleic acid amplification method that can eliminate the need for repeated temperature cycling. Ideally, all sequences in the target set will match the primers used for amplification exactly, while none of the sequences in the background set will match the primers. Therefore, oligonucleotide specificity is one of the most critical factors for efficient PCR; optimal primers should hybridize only to the target sequence, especially when complex genomic DNA is used as the template. Alternative product amplification can also occur when primers are complementary to inverted repeats and produce multiple bands. These amplification problems often occur when primers anneal to interspersed repeats (tandem repeats or retrotransposons). Inter-repeat amplification polymorphism techniques for eukaryotes have exploited highly abundant dispersed repeats, such as long terminal repeats (LTRs of retrotransposon sequences). The association of these sequences allows amplifying a set of bands (DNA fingerprints) using primers homologous to these high copy number repeats. The PCR products, and thus the fingerprint patterns, result from the amplification of hundreds to thousands of target sites in the genome. Primers complementary to repetitive DNA can produce many non-specific bands in single-primer amplification products and interfere with the performance of the target PCR. However, the generic DNA fingerprinting methods are based on generating sequences of inverted repeats. Often, only one primer is used in this PCR; the ends of the products should consist of an inverted repeat complementary primer sequence. The genomes of many prokaryotes and eukaryotes have been sequenced and annotated in databases. For this reason, *in silico* approaches are becoming widely used to extract useful information from input data sets and to process them further using virtual tools to prepare and predict experimental results in the design phase. One such approach is virtual (*in silico*) PCR. *In silico* PCR tests the target location and amplicon size in one or more DNA templates. Although the primary goal of *in silico* PCR is to predict the expected products upon amplification of the DNA template with the specified primer set, related tasks commonly used by researchers include primer or probe searching, target location, oligonucleotide design, and analysis such as evaluating the melting temperature of primer pairs. Currently, several web-based methods for *in silico* PCR have been implemented. Electronic PCR is a web server that allows heuristic searches of predefined genomes with up to two mismatches. UCSC *In-Silico* PCR (http://genome.ucsc.edu/cgi-bin/hgPcr) is a web server that uses an undocumented algorithm to search a predefined genome (Nassar et al., 2023). Primer-BLAST (https://www.ncbi.nlm.nih.gov/tools/primer-blast/) is a web server that uses BLAST as its underlying search method (Ye et al., 2012). It should be noted that they are all not available as stand-alone software, except for our FastPCR software (Kalendar et al., 2011; Kalendar et al., 2017). Furthermore, adapting some commonly used sequence similarity search methods to *in silico* PCR is not optimal or effective. Therefore, an additional task for *in silico* PCR is identifying multiple binding sites, including mismatched hybridization, which is usually performed by considering the similarity of the primer to the targets across the entire primer sequence. In addition*, in silico* PCR software must also handle multiplexed, nested, or tilling PCR, an approach commonly used to amplify multiple DNA target regions in a single reaction (DNA fingerprinting) (Arvas et al., 2023). Virtual PCR is performed by a computer program with an input of a pair or batch of primers against the sequence(s) under study or an intended genomic sequence (Abileva et al., 2024). *In silico* PCR aims to test the specificity of the PCR application, including the target location and amplicon size in one or more target genome(s). Therefore, the use of primers is not limited to PCR nucleic acid amplification but extends to all standard molecular biology methods. These considerations motivated the development of a high-throughput, non-heuristic algorithm implemented as stand-alone Java software with a command-line interface that incorporates virtual PCR capabilities. In developing the Java tool, we aimed to create a practical, efficient, and easy-to-use software for multiple primer or probe searches for linear and circular DNA sequences. We also aimed to predict amplicons via *in silico* PCR from large or small local databases. This *in silico* tool is useful for quickly analyzing primers or probes against target sequences, determining primer location, orientation, and binding efficiency, and calculating the melting temperature (Tm) for primer-template duplex. This tool allows the isolation and characterization of sequences in genomic DNA using degenerate primers and determining the copy number of the target amplicons. It is useful for validating existing primers, probes, and their combinations. PCR products can likely be found for linear and circular templates using standard or inverse PCR and DNA fingerprinting.

## Methods

### Plant material and DNA extraction

Grains of maize line Mo17 were kindly provided by the Maize Research Section, Agricultural Research Center (ARC), and the U.S. Department of Agriculture (USDA). DNA was isolated from leaves of 10-day-old seedlings using the SDS-proteinase K column protocol (Kalendar et al., 2023). DNA samples were diluted in 1×TE solution (0.1 mM EDTA, 10 mM Tris-HCl, pH 8.0), and the total DNA concentration was determined using a NanoDrop1000 spectrophotometer (Thermo Fisher Scientific Inc.).

### IRAP-PCR analysis

Thirty LTR primers were designed based on the most abundant LTR retrotransposons in maize (Cinful1 (AC231746), Huck1 (AC230001), Ji (DQ002406), Opie (AY664413), Grande (AY664416.1:70909-83340), and Tekay (AF050455)) (Ghonaim et al., 2020). The selected primers matched the motifs sufficiently conserved in the retrotransposons to allow amplification of the vast majority of targets in the genome.

The IRAP amplification was performed according to (Kalendar et al., 2020), using 30 primers for these LTR retrotransposons. PCR was performed in 25 µL reaction mixtures containing 25 ng DNA, 1× DreamTaq buffer, 200 mM dNTP, 400 nM primer, and 1 U DreamTaq DNA Polymerase (Thermo Fisher Scientific). Amplification was performed on a SimpliAmp™ Thermal Cycler (Thermo Fisher Scientific Inc.). The PCR reaction program consisted of 1 cycle at 95°C, 2 min; 30 cycles of 95°C for 15 s, 60°C for 20 s, 72°C for 40 s; and a final extension at 72°C for 2 min. PCR products were separated by electrophoresis at 70 V for 5 h in a 1.4% agarose gel with 1 × TBE buffer (Tris-Borate-EDTA buffer, pH 8.3) electrophoresis buffer. Gels were stained with ethidium bromide (EtBr) and visualized on a 1% agarose gel using the ChemiDoc XRS + Gel Imaging System (Bio-Rad Laboratories, Inc.).

### The search algorithm

The principal objective of the algorithm is to facilitate the efficient identification of complementary sequences within the template DNA, with a maximum permitted number of mismatches. The sequences in question must be situated at a specific distance from one another, which corresponds to the maximum size of the anticipated PCR product. Stable hybridization of a primer to template DNA is a prerequisite for primer extension by DNA polymerase. Mismatches impact the stability of the primer-template duplex and the efficiency with which the polymerase extends the primer. While any mismatch will impact PCR specificity, mismatches at the 3′end of a primer have a pronounced negative effect on primer extension. A two-base mismatch at the 3′end of the primer will result in PCR failure. Accordingly, the algorithmic approach in question accords particular attention to the 3′end of the primer, calculating its degree of similarity to the template with a user-defined level of stringency. A limited number of mismatches can be permitted, typically at the expense of reduced amplification efficiency. Consequently, the algorithmic approach has been refined by incorporating data derived from actual PCR experiments, specifically regarding how mismatches are handled.

**FIGURE 1:**
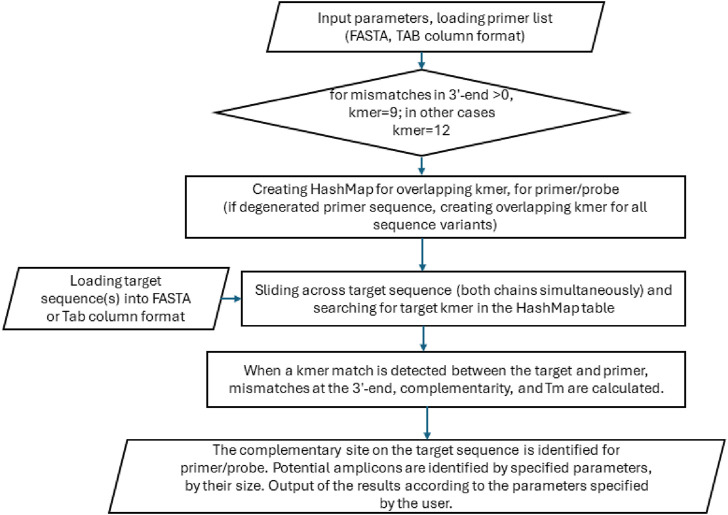
Workflow for the main steps of the software to execute *in silico* PCR.

The algorithm is comprised of three distinct components (Figure 1). The initial stage of the process entails the creation of a HashMap table consisting of a primer set’s overlapping k-mers [defined as words of a fixed length (k)]. The second component of the algorithm performs sequence analysis using the hash above table. The third component is responsible for predicting potential PCR products for linear or circular templates, should this be a requisite of the user. The hash table for all overlapping k-mers is stored in memory as a map structure, comprising a list of k-mers and indexes to a supplementary array. The assisting array is linked to a specific primer and kmer coordinate, thus enabling the identification of analogous, identical, or repeated nucleotide sequences within the primer set. The length of the k-mer may be either 9 or 12 nt, contingent on the desired level of sensitivity, the nature of the task at hand, and the length of the primers. Notably, employing a long k-tuple of 12 nt does not result in a loss of sensitivity or false negatives, as the algorithm permits up to one mismatch in each kmer. The capacity of our algorithm to accommodate mismatches, in conjunction with its compatibility with degenerate nucleotides and its ability to detect stable guanine mismatches (i.e., G-G, G-T, and G-A), distinguishes it from other published approaches, including BLAST. Furthermore, our algorithm permits the utilization of short primers (12 nt). The input DNA sequence may contain degenerate nucleotides that are accepted by the IUPAC code, including M (A/C), R (A/G), W (A/T), S (G/C), Y (C/T), K (G/T), V (A/G/C), H (A/C/T), D (A/G/T), B (C/G/T), and N (A/G/C/T).

The algorithm employs a sliding window approach to identify primer target sites, traversing the DNA sequence at one-nucleotide intervals. The algorithm does not construct a hash table for the template sequence; instead, it continually attempts to identify matches by utilizing the primer hash table. Individual kmers are extended in both directions until a region of similarity is identified or until a critical number of mismatches is reached. It should be noted that both the primer and target sequences can contain degenerate nucleotides. The user can specify the number of mismatches that may occur near the 3′end of the primer, with a default value of one mismatch permitted in the last seven nucleotides. The adjustable 3′mismatch threshold makes the algorithm compatible with oligonucleotide hybridization probes (molecular beacons) that lack complementarity to the target at their 3′ends.

The user has the option of defining the desired size of the PCR product. The default distance between the forward and reverse primers is 50 to 5,000 bases. Predicting PCR product size is feasible for both linear and circular templates, employing standard, inverse, or multiplex PCR and bisulfite-treated DNA as a template. The algorithm can accept single or multiple DNA sequences as primers or amplification targets.

*In silico* PCR experiments with oligonucleotides that mismatched their targets were conducted, and the primer melting temperatures (Tm) were calculated using the averaged nearest neighbor thermodynamic parameters (Shing Ho, 1994; SantaLucia et al., 1996; Peyret et al., 1999; Lane et al., 2008). The PCR annealing temperature (Ta) was calculated as the value for the primer with the lowest Tm plus the natural logarithm of the PCR product length.

## Results

### *In silico* PCR analysis

We developed an easy-to-use command line Java application that can identify potential PCR amplicons or sequences that can be applied to any target genome (https://github.com/rkalendar/virtualPCR). This tool is implemented in Java (requires Java Platform, Standard Edition 22 or higher) and does not require additional steps, such as the individual compilation of certain blocks for a particular operating system or the installation of additional libraries, as it is a standard application for the Java Platform.

To validate the software, the NCBI’s Genome resource of genome sequences (https://www.ncbi.nlm.nih.gov/genome/) was used as a target set to measure search performance and accuracy (false negatives and false positives), and primer and probe sequences from the retrotransposon sequences obtained as primer sets. The time and memory were tested using an HP Z6 G5 machine [Intel(R) Xeon(R] Silver 4215R CPU 3.20 GHz, 3.19 GHz (2 processors), 64.0 GB, Windows 11 Pro (23H2) by running the software using complete genomic DNA sequences of different sizes with a list of primers with standard and degenerate bases both for the specified primer and target.

The tool’s algorithm saves memory by creating a hash table only for primer sequences and is computationally efficient, approaching a linear time in database size. The tool’s memory and execution time requirements were determined using genomic DNA sequences of different sizes and primer sequences with standard or degenerate bases. Because the entire DNA sequence was loaded into the RAM, the memory required for analysis was directly proportional to the sequence length (Figure 1).

The software is compatible with single-primer/probe methods based on sequence repeats such as SINE-PCR (Miller and Archibald, 1993), Inter-retrotransposon amplified polymorphism (IRAP) (Kalendar and Schulman, 2006), inter simple sequence repeats (ISSRs), and random amplified polymorphic DNA (RAPD) (Williams et al., 1990), as well as methods utilizing multiple primers, such as LAMP (Notomi et al., 2000) and multiplex PCR. It is also compatible with retroelement-based genotyping and DNA fingerprinting methods. The primer and probe lengths can be set between 12 and 500 nucleotides, and the maximal PCR product (amplicon) length is limited only by polymerase processivity and not by software. The analysis results included PCR product sequences, lengths, and Ta values presented separately for nuclear, plastid, and mitochondrial DNA. Another software application detects plasmid sequences based on the ends of an inserted sequence. For this purpose, sequence fragments flanking an insertion should be used instead of primer sequences, and the “probe search” option should be selected in the software.

### *In silico* PCR application for transposable element identification

To demonstrate the capabilities and potential of our software, we performed *in silico* PCR analysis of several complete plant and animal genomes using a list of primers corresponding to an inverted repeat sequence of *Hordeum-Triticum Athos* miniature inverted-repeat transposable element (MITE) sequences (Feschotte and Mouches, 2000). MITE nonautonomous members of Class II element families are derived by internal deletion of autonomous elements; these members are short (70–300 bp in length) and have conserved terminal repeats. For example, *Athos*, is a MITE families described in grasses (Ubi et al., 2022). Using Blast for the RefSeq Genome Database (GCF_904849725.1), we collected Athos element sequences from the genome of Hordeum vulgare, of which there were 205 per complete genome. *Athos* element sequences are highly truncated, including partial loss of terminal inverted repeats in the barley genome. Sequences of terminal inverted repeats contain multiple point mutations, insertions, or deletions, which causes difficulty in selecting universal primers that cover all whole copies of a particular *Athos* element. Therefore, we selected all unique sequence variants for terminal inverted repeats and used them as a list of 46 primers to identify and obtain complete MITE elements for the genomes of other cereals. The human and long-horned nomad bee genome (*Nomada hirtipes*) was used as a negative control. Since the sequences of terminal inverted repeats for the Athos element were different and quite degraded, we used all 46 unique variants simultaneously as Forward primers in the analysis. The same primers were used for forward and reverse amplification. All the primers were 15 nucleotides long and located in the most distant region of the terminal inverted repeat in the *Athos* element. The size of the amplicon, in this case, could be 30 to 200 (possibly up to 300) nucleotides, including truncated elements with a central region. We used the following search conditions with control options: type = primer number3errors = 0; minlen = 30; and maxlen = 200. The results of this analysis are presented in Table 1. In the genome of *Hordeum vulgare*, 768 *Athos* and related elements were detected, which was much more significant than detected by Blast analysis (205 copies for GCF_904849725.1, RefSeq Genome Database). We detected *Athos* elements and related MITE elements with identical terminal repeats (Table 1). In our analysis, we could detect only the whole Athos and associated elements that contained both repeats, whereas the central part could vary. Concerning the genome of *Hordeum bulbosum*, 1,620 records of complete *Athos* and related elements were detected compared to those of other species of the *Hordeum* family. This corresponds to the doubled genome size of this species compared to other species of the *Hordeum* family. For wheat genomes (*Aegilops tauschii*, *Triticum dicoccoides*), which are the most similar to species of the *Hordeum* family, numerous copies of the related Athos element were detected, with this MITE occurring much more frequently in the wheat genome than in the genome of *H. vulgare*. The copy number of *Athos* and related elements directly depends on the genome size; the more significant the genome is, the more copies of this element are detected.

**TABLE 1:** Software execution times and total hits were returned by searching several eukaryotic genomes using a list of 46 primers corresponding to an inverted *Athos* MITE sequence repeat.

Genome	GenBank assembly	Genome size (total number of files)	30–181 bp amplicons limit (30–200 bp)	Time in seconds
*Hordeum vulgare*	GCA_904849725.1	4.2 Gb (8)	768	152
*Hordeum marinum*	GCA_022496015.1	3.8 Gb (7)	631	127
*Hordeum bulbosum*	GCA_963506655.1	7.3 Gb (14)	1,620	259
*Aegilops tauschii*	GCF_002575655.2	4.2 Gb (8)	953	152
*Triticum dicoccoides*	GCA_002162155.3	10,7 Gb (14)	1922	418
*Oryza sativa*	GCA_001433935.1	373.8 Mb (11)	613	11
*Zea mays*	GCA_902167145.1	2.2 Gb (12)	286	70
*Brachypodium distachyon*	GCA_000005505.4	271.2 Mb (6)	590	8
*Chenopodium album*	GCA_948465745.1	1.6 Gb (28)	199	48
*Capsicum baccatum*	GCA_030864225.1	3.3 Gb (12)	37	92
*Helianthus annuus*	GCA_002127325.2	3 Gb (17)	7	62
*Cycas panzhihuaensis*	GCA_023213395.1	10.5 Gb (11)	0	284
*Nomada hirtipes*	GCA_951802735.1	316.5 Mb (17)	0	1
*Homo sapiens*	GCF_000001405.40	3.1 Gb (25)	0	89

The genomes of *Oryza sativa*, *Brachypodium distachyon*, and *Zea mays*, which are genetically distant from species of the *Hordeum-Triticum* family, exhibited a high frequency of occurrence of related Athos and related elements (613 copies in the genome of *Oryza sativa*, and 590 copies in the genome of *B. distachyon*). Sequence analysis of the *Athos* element in these genomes revealed that the sequences obtained corresponded to the structure of MITE families with complementary inverted repeats characteristic of this type. Thus, in the genomes of *Oryza sativa*, *B. distachyon*, and *Z. mays*, new MITE families related to the Athos element in the genome of the Hordeum family were identified. Identifying related MITE elements for genetically distant plant species is expected to be problematic. However, sequences structurally consistent with MITE elements have been identified for the genomes of *Capsicum baccatum*, *Chenopodium album*, and *Helianthus annuus* from the class *Dicotyledones*. Thus, this approach makes it possible to locate MITE elements in closely related species and genetically arranged species belonging to different plant classes (*Monocotyledones - Dicotyledones*) from the common genus *Angiosperms*. Meanwhile, no sequences corresponding to MITE elements were detected in this analysis for the genome of *Cycas panzhihuaensis*, which belongs to *Gymnosperms* (Class *Cycadopsida*) divisions. As expected, no amplicons or long-horned nomad bees were predicted for the human genome due to the lack of *Athos* families in animals.

### *In silico* PCR application - virtual genome fingerprinting

**FIGURE 2:**
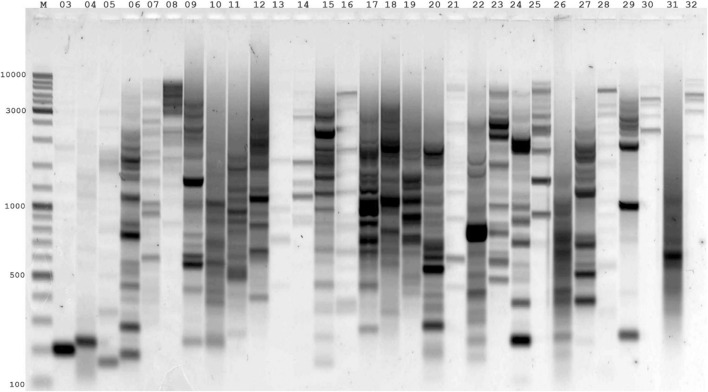
Agarose gel electrophoresis patterns of genome fingerprinting products amplified via IRAP-PCR for individual LTR primers of the maize line (Mo17). The numbering of LTR primers (4,303–4,332) corresponds to the number 03–32. LTR primers (4,303–4,307) correspond to the LTR retrotransposon (*Huck*); LTR primers (4,308–4,312) correspond to the LTR retrotransposon (*Ji*); LTR primers (4,313–4,319) correspond to the LTR retrotransposon (*Opie*); LTR primers (4,320–4,324) correspond to the LTR retrotransposon (*Grande1-4*); LTR primers (4,325–4,330) correspond to the LTR retrotransposon (*Tekay*); LTR primers (4,331–4,332) correspond to the LTR retrotransposon (*Cinful1*). M - Thermo Scientific GeneRuler DNA Ladder Mix (100–10,000 bp).

Another valuable task using virtual genome fingerprinting is analyzing the use of a single LTR primer to identify nested LTRs for genotyping. This task requires the preparation of a list of effective primers for use in a particular genome and analyzing the use of a single LTR primer in related genomes. In classical *in silico* PCR, the necessary condition for an amplicon search is two primer-binding sites on complementary DNA strands located at a certain distance and orientation relative to each other. IRAP uses a single primer complementary to a conserved LTR site on a retrotransposon. Suppose that a specific multicopy LTR-retrotransposon in the genome under study is distributed throughout the genome, forming closely neighboring LTR fragments. In that case, PCR can detect these genes with a single primer. Conserved LTR primers can be used for closely related genomes to genotype a particular LTR retrotransposon in another genetically similar species. However, first, it is necessary to assess the potential of each LTR primer or to identify the LTR retrotransposon sequence in the genome of another species. Our tool can be used for virtual genome fingerprinting and identifying new sequences of LTR retrotransposons. For virtual genome fingerprinting, we used a list of LTR primers obtained for several LTR retrotransposons (*Huck*, *Ji*, *Opie*, *Grande1-4*, *Tekay*, and *Cinful1*) in the *Z. mays* genome (Ghonaim et al., 2020). All the LTR primers used in the IRAP were used to determine the efficiency of the genome fingerprinting of the breeding accessions of this species (Figure 2; Supplementary Table S1). The following search conditions were used for the control options: type = primer number3errors = 0; minlen = 100; and maxlen = 3,000. The results of this analysis are presented in Supplementary Table S1. A comparative study of wet laboratory and virtual genome fingerprinting showed a convincing correlation between the obtained theoretical and practical data. According to virtual genome fingerprinting analysis, LTR primers that formed low abundance amplicons showed low abundance PCR products when amplified from *Z. mays* samples via wet laboratory experiments. The genetically similar genomes of *B. distachyon*, *Oryza sativa*, and *T. dicoccoides* were used as controls for LTR primers, which showed the highest occurrence in the *Z. mays* genome. As a result of the analysis, no amplicons were detected for these genomes. This result shows the algorithm’s sensitivity to false positives, and no sequences of LTR primers from the *Z. mays* genome were detected.

DNA polymerases with and without proofreading functions had different effects on the extension of 3′terminal mismatched primers. The proofreading DNA polymerases efficiently removed the mismatched base from the 3′terminal mismatched primer. Polymerases without proofreading activity do not require stringent base pairing at the initial step of primer extension. This explains the presence of PCR amplification products during RAPD-like amplification for most primers at low annealing temperatures and high primer concentrations. The differences between the single-base discrimination abilities of polymerases with and without proofreading function affect the sensitivity and specificity of any PCR variant. Since genome fingerprinting is performed with polymerases without proofreading activity (Taq-polymerase) at high primer concentrations and low annealing temperatures, PCR products with DNA templates from 3′terminal mismatched primer can be amplified (Zhang et al., 2003; Kalendar et al., 2022). Usually, such PCR products are detected during the last PCR cycle. In contrast, detecting amplicons with entirely complementary primers occurs much earlier than other methods and does not require additional amplification cycles. Our study showed that several LTR primers in the *Z. mays* genome produced weaker PCR amplicons. In virtual genome fingerprinting for these LTR primers, a small number of amplicons, no more than 10 per genome, is expected. For LTR primers for which a large number of products (more than 50) are expected in virtual genome fingerprinting, we observe very intensive amplification with a large number of bands and the effect of overamplification (the formation of the main product ended many cycles earlier, and in the current cycles there is concatemerization of amplicons). Thus, the results obtained by virtual genome fingerprinting correlate perfectly with those of PCR experiments before IRAP genome fingerprinting of the *Z. mays* genome.

### *In silico PCR* application, for example, with six genes for the human genome

We performed *in silico* PCR analysis to detect PCR products for the human genome (genome assembly GRCh38. p14). Six genes from different human chromosomes and different lengths were taken into the study: CA2, COL1A1, COL1A1, FAM20, BEST4, SHF, and MMP9 (Supplementary Data). The primer detection parameters were default, one error at the 3′-end of the primer was allowed, and the PCR product size did not exceed 1 kb. Additionally, the software extracted the amplicon sequence from the chromosome. The specificity and efficiency of each PCR pair to the target genes were performed *in vitro* by PCR with human DNA. This PCR analysis confirmed the correct length of each target amplicon and specificity for the human genome (single PCR product). Thus, the laboratory results confirmed the efficiency of the algorithm used for *in silico* PCR analysis. PCR analysis revealed only specific sequences for target genes in the human genome, amplicons’ expected size, and chromosomal localization. No non-specific PCR product was detected for all target genes in the human genome in our proposed *in silico* PCR algorithm. In addition, PCR primers were designed for the previous human genome assembly (GRCh37. p13). If there were problems with this assembly for specific genes, there could also be problems with *in silico* PCR for the later human genome assembly. No issues, however, were identified. Complete reports on *in silico* PCR analysis for each primer pair for each target gene are available at the Zenodo digital library (https://zenodo.org/records/13625500).

### *In silico* PCR application for isolating and characterizing plant copia-type reverse transcriptase fragments

The efficiency of our proposed algorithm was validated for the isolation and characterization of reverse transcriptase (RT) fragments from different genomes using degenerate primer pairs. The RT gene of retroelements has several conserved domains characteristic of individual retroelement families. The availability of degenerate oligonucleotide primers complementary to the conserved regions of RT allowed amplification of RT fragments and sampling of retrotransposon diversity in several plant genomes (Kolano et al., 2013). Several plant genomes, fungi, and, as control genomes, insects, animal, and human genomes were used for the analysis. The Ty1-copia degenerate primer pair corresponds to the highly conserved peptide sequence of the reverse transcriptase of the Ty1-copia group retrotransposons, ubiquitous components of plant genomes. In this case, the length of the amplicons was 270–281 bp, which is the expected value.

>RT+(QMDVK).

5′-CARATGGAYGTNAARAC.

>RT-(YVDDML)

5′-CATRTCRTCNACRTA.

The following *in silico* search parameters were used: number3errors = 1, minlen = 200, and maxlen = 500. This corresponded to an amplicon size between 200 and 500 bp and allowed a non-complementary base at the 3-terminus of the primer. The command - SequenceExtract = true was also used to obtain the sequences of each RT fragment. During the analysis, the summary information, including amplicon sequences, their length, and Ta, is presented separately for each target file. For each chromosome for each of the genomes studied, the resulting RT fragments were aligned using the MAFFT multiple sequence alignment (MSA) tool (Rozewicki et al., 2019). Only RT fragments detected by our method were used to approximate the number of RT fragments for a particular chromosome and plant genome. Unique RT fragments were analyzed using the Blast tool for reference genomes. Using unique RT fragments to explore a particular plant genome is to obtain the likeliest number of copies of a specific Ty1-copia retrotransposon. If the sequences are similar, several duplicate matches for different RT fragments will be observed for one location in the genome. The parameters for identification of RT fragments included number3errors = 1, which allowed the software to ignore the non-complementary nucleotide at the 3-terminus of the primer. Parameters excluding the non-complementary nucleotide at the 3-terminal primer (number3errors = 0) detected the reduced number of detectable RT fragments in the plant genome. Therefore, we focused on the parameter number3errors = 1 for all studied genomes, plants, animals, fungi and insects. Animal and fungal genomes were used as control genomes in which, theoretically, the RT primers used should not be effective in identifying reverse transcriptase fragments for Ty1-copia plant retrotransposon. However, the parameters used for *in silico* PCR analysis for animal and *Hydra vulgaris* genomes identified amplicons larger than expected for plant RT fragments. Thus, for the *Homo sapiens* and *H. vulgaris* genomes, the identified amplicons were most likely false positives; however, for the *Rattus norvegicus* genome, most of these amplicons were repetitive for diverse regions of the genome. We cannot confirm that these amplicons from the *R. norvegicus* genome are related to reverse transcriptase. Still, their frequency of occurrence in the genome is also not random and may have something to do with genome repeats, viral or other in origin. In addition, 13 amplicons were identified for the insect genome (*N. hirtipes*), and only 2 amplicons were false positives (233, 351 bp). In contrast, the other amplicons were directly related to the coding part of the retrotransposons. Another interesting fact about this genome is that the amplicons obtained corresponded to the size expected for plant RT fragments (276–281 bp). For the fungus’s genome (*Puccinia triticina*), we identified 41 amplicons, most of which were 384 bp, which is not typical of what is expected in plant RT fragments. However, we identified that all of these amplicons (41) matched the homologous sequence of the reverse transcriptase fragments. Thus, *in silico* PCR analysis using degenerate RT primer pairs allowed the isolation and characterization of reverse transcriptase fragments from fungi and insect genomes. Therefore, this approach can be extended by using it to isolate and characterize conserved reverse transcriptase fragments for genomes genetically distant from plants. The application of options for *in silico* PCR analysis using degenerate primer pairs, which restrict non-complementary nucleotides at the 3-terminus of the primer but which are allowed at the 5-terminus upstream of the central part of the primer, prevents false-positive amplicons detection and reduces the total number of detectable RT fragments for plant genomes. The analysis results of the plant, animals, and other genomes are shown in Table 2 and Supplementary Table S1. Complete reports on *in silico* PCR analysis for each primer pair for each target gene are available at the Zenodo digital library.

**TABLE 2:** Comparative analysis of *in silico* PCR application for isolating and characterizing plant Copia-type reverse transcriptase fragments and NCBI BLAST.

Tool	Total number of matches	Number of false positive matches
*Arabidopsis thaliana*		
NCBI BLAST	38	0
*In silico* PCR tool	62	0
*Brachypodium distachyon*		
NCBI BLAST	312	0
*In silico* PCR tool	509	0
*Zea mays*		
NCBI BLAST	37 651	0
*In silico* PCR tool	39 710	0
*Capsicum annuum*		
NCBI BLAST	6 246	0
*In silico* PCR tool	3 747	0
*Hordeum vulgare*		
NCBI BLAST	43 939	0
*In silico* PCR tool	41 781	0
*Triticum dicoccoides*		
*In silico PCR tool*	75 358	0
*Vitis vinifera*		
*In silico PCR tool*	913	0
*Puccinia triticina* (fungi)		
*In silico PCR tool*	41	0
*Nomada hirtipes* (insect)		
*In silico PCR tool*	13	2
*Hydra vulgaris*		
*In silico PCR tool*	7	7
*Rattus norvegicus* (mammals)		
*In silico PCR tool*	24	24
*Homo sapiens* (mammals)		
*In silico* PCR tool	11	11

## Comparison with other software packages

Several web-based software tools are available for *in silico* PCR analysis. The most commonly utilized tools include NCBI/Primer-BLAST (http://www.ncbi.nlm.nih.gov/tools/primer-blast/) (Ye et al., 2012), and web server “*In silico* simulation of molecular biology experiments” (http://insilico.ehu.es/) (Bikandi et al., 2004; San Millan et al., 2013), WebPCR (https://pydna.pythonanywhere.com/) (McCann, 1999) and UCSC In-Silico PCR (http://genome.ucsc.edu/cgi-bin/hgPcr) (Nassar et al., 2023) are worthy of mention. Table 3 presents a comparative analysis of these web-based software tools with our software. Compared to our software, other web-based software only extends beyond the classical PCR approach with two primers. For example, BLAST generates local alignments that may not cover the entire primer sequence, it does not support searching for query pairs separated by arbitrary sequences of variable length, degenerate primers cannot appear in alignment seeds and therefore require special handling to avoid further false negatives, and parameters sensitive enough to find an acceptable number of matches tend to generate large numbers of false positives. Significant post-processing is required to identify valid hits. Primer-BLAST uses a word length of 7, so sites must have an identical 7-mer match to a primer (this can lead to false negatives). In silico PCR software must also handle degenerate primers or probes, including those with 5′or 3′tail sequences and single nucleotide polymorphisms (SNPs). Bisulfite-treated DNA was used as the template because it contains no cytosine other than the methylated cytosine in a CG dinucleotide. In addition, a complete genomic analysis of primers complementary to repetitive elements in the genome is required. NCBI/Primer-BLAST is incapable of searching for pairs of queries separated by arbitrary sequences of variable length, is incompatible with degenerate nucleotide sequences, does not consider sequence repeats, does not accept one or several primers, does not allow the use of primers with the same sequence, and is incompatible with methods employing molecular beacons. The maximum length of the primer was limited to 36 nt, which is not a limitation in our software. NCBI/Primer-BLAST can only locate and display the most remote sites if the primer has multiple binding sites at repeated sequences. Furthermore, the primer order was fixed in NCBI/Primer-BLAST; forward and reverse primers were entered according to the following criteria: “forward primer (5'->3′on the plus strand)” and “reverse primer (5'->3′on the minus strand)”.

**TABLE 3:** Comparison of several *in silico* PCR tools.

Features	VirtualPCR tool	Primer-BLAST	The web server*: In silico* experiments with complete genomes)	WebPCR
Input				
Whole genomes/maximal length of PCR template	+/any	+/50000	+/−	−/any
Multiple templates	+	−	−	−
Linear/circular templates	+/+	+/−	+/−	+/−
Bisulfite modification compatibility	+	−	−	−
Degenerate oligonucleotides in primer and template	+	−	−	−
Accepted number of primers	any	2	2	2
Minimal/maximal primer length	12 to any	15/36	10/35	10/35
Searching				
Singleplex/multiplex primers and probes	+/+	+/−	+/−	+/−
Mismatches accepted within primer and target	+	+	−	−
Addition of non-complementary sequences at one or both termini of probe and primer	+	−	−	−
Linked (associated) sequences searching	+	−	−	−
Exon/intron selection	−	+	−	−
DNA fingerprinting	+	−	+	−
*In silico* PCR for techniques based on repeats (SINE-PCR, IRAP, ISSR, RAPD, etc.)	+	−	−	−
BLAST online search with genome database	−	+	−	−
Output				
Graphical interface	−	+	+	+
Annealing and melting temperatures were calculated with standard and degenerate oligonucleotides for oligonucleotide-target duplexes	+	−	−	−
Search for all variants of amplicons within repeated sequences	+	−	−	−
List of potential amplicons	+	−	−	−
Other				
Primer design parameters validated in the lab	+	+	+	+
High-throughput compatibility	+	−	−	−

+ supported feature, −unsupported feature.

Our software is fully compatible with PCR fingerprinting techniques based on sequence repeats, such as the random amplified polymorphic DNA (RAPD) technique. Single or multiple primers can amplify hundreds to thousands of inter-repeat DNA sequences for genome fingerprinting. The software can rapidly search for primers or probes and determine their location, orientation, melting temperature, and binding efficiency. Furthermore, the current primers, probes, and combinations of primers can be validated. The software is capable of handling degenerate nucleotides in primers, probes, and target sequences and can predict PCR products from both linear and circular templates using standard or inverse PCR and multiplex PCR. Therefore, our tool is not limited to canonical PCR but applies to various other PCR-based methods, including potential future refinements and advancements.

## Conclusion

In this study, we present the development and validation of a DNA sequence analysis algorithm for a range of experimental scenarios, including, but not limited to, using two primers in a standard PCR procedure. The core algorithm is relatively simple, comprising only a few hundred lines of Java code and exhibiting the potential for parallelization. An indispensable algorithm component is genome fingerprinting, which enables sophisticated searches for single or multiple primers on a genome scale. The algorithm is compatible with sequences as short as 12 nt, allowing the users to search for short conserved sequences, including promoters and other regulatory elements. Moreover, modifications to the search criteria can be readily implemented, ensuring the algorithm’s compatibility with emerging methodologies and providing a future-proof solution. The algorithm’s efficacy was validated by identifying MITE transposons in diverse plant and animal genomes. The results of the software validation showcased the ability to perform intricate computational operations that extend beyond the scope of conventional *in silico* PCR. For instance, *in silico* PCR analysis using degenerate primer pairs for plant Ty-copia-type reverse transcriptase fragments allowed the isolation and characterization of reverse transcriptase fragments from fungi and insect genomes. Our software demonstrated its suitability for tasks where other freely available software would be inadequate or require extensive post-processing of output data.

## Data Availability

The datasets presented in this study can be found in online repositories. The names of the repository/repositories and accession number(s) can be found in the article/Supplementary Material. The article contains data supporting this work, and the online supplementary material and data supporting this study's results are available at Zenodo digital library (https://zenodo.org/records/13625500). The virtualPCR software (https://github.com/rkalendar/virtualPCR) was written in Java and requires a standard Java Runtime Environment (JRE 22 or above). The online web server version of the application is available on the website: https://primerdigital.com/tools/epcr.html.

## Supplementary material

SUPPLEMENTARY TABLE S1
PCR analysis of primer pairs for human genes in comparison with *in silico* PCR results.

SUPPLEMENTARY TABLE S2
List of LTR and MITE primers used.

TABLE S1
Comparison of *in silico* PCR tool with amplicon analysis (Number of Matches) from NCBI BLAST.
