# Supplementary material for: In silico PCR analysis: a comprehensive bioinformatics tool for enhancing nucleic acid amplification assays
Source: Front Bioinform. 2024 Oct 7;4:1464197. doi: 10.3389/fbinf.2024.1464197 (PMC11491563; doi:10.3389/fbinf.2024.1464197)
Supplement: Supplementary file 2 [file DataSheet1.docx]

**Supplemental Data**

***In silico* PCR analysis: a comprehensive bioinformatics tool for enhancing nucleic acid amplification assays**

Ruslan Kalendar **^1,2*^**, Alexandr Shevtsov **^3^**, Zhenis Otarbay **^4^**, Aisulu Ismailova **^5^**

^1^ Helsinki Institute of Life Science (HiLIFE), University of Helsinki, Biocentre 3, P.O. Box 65, Viikinkaari 1, 00014, Helsinki, Finland

^2^ National Laboratory Astana, Nazarbayev University, 53 Kabanbay Batyr Ave., 010000, Astana, Kazakhstan

^3^ National Center for Biotechnology, 13/5, Kurgalzhynskoye Road, 010000, Astana, Kazakhstan

^4^ Astana IT University, Prospekt Mangilik Yel., 020000, Astana, Kazakhstan

^5^ S. Seifullin Kazakh Agrotechnical Research University, Department Information Systems, Zhengis Ave 62, 010000, Astana, Kazakhstan

***Correspondence**:

Ruslan Kalendar, [ruslan.kalendar@helsinki.fi](mailto:ruslan.kalendar@helsinki.fi), phone: +358294158869

R.K.: [ruslan.kalendar@helsinki.fi](mailto:ruslan.kalendar@helsinki.fi) ORCID 0000-0003-3986-2460

A.S.: [shevtsov@biocenter.kz](mailto:shevtsov@biocenter.kz) ORCID 0000-0002-0307-1053

Z.O.: [zhenis.otarbay@nu.edu.kz](mailto:zhenis.otarbay@nu.edu.kz) ORCID 0000-0003-4111-0719

A.I.: [a.a.ismailova@kazatu.kz](mailto:a.a.ismailova@kazatu.kz) ORCID 0000-0002-8958-1846

**Data availability and implementation**

The article contains data supporting this work, and the online supplementary material and data supporting this study's results are available at Zenodo digital library (<https://zenodo.org/records/13625500>). The virtualPCR software (<https://github.com/rkalendar/virtualPCR>) was written in Java and requires a standard Java Runtime Environment (JRE 22 or above). The online web server version of the application is available on the website: <https://primerdigital.com/tools/epcr.html>

**PCR analysis of primer pairs for human genes in comparison with *in silico* PCR results**

**Table S1.** PCR primers used for gene amplification in humans.

| **ID** | **Sequence (5'-3')** | **nt** | **Tm (°C)** | **GC%** | **Amplicon size (bp) / Tm(°C)** | **Topt**  **(°C)** | **GeneID** | **Genome location (**Homo sapiens**)** |
| --- | --- | --- | --- | --- | --- | --- | --- | --- |
| 5836 | gtgacatcatacttaacgctgca | 23 | 61.3 | 43.5 | 758/79 | 67 | CA2:NM_001293675:exon3:c.T78G:p.Y26X | 86386182-86386981 chromosome 8, GRCh37.p13 Primary Assembly |
| 5837 | agtactgtctagcctcaggttaa | 23 | 60.5 | 43.5 |  |  |  |  |
| 5838 | gtctccatgttgcagaagacttt | 23 | 61.3 | 43.5 | 657/89 | 68 | COL1A1:NM_000088:exon48:c.A3659T:p.D1220V | 48263756-48264556 chromosome 17, GRCh37.p13 Primary Assembly |
| 5839 | gcaaagatggactcaacggtc | 21 | 62.1 | 52.4 |  |  |  |  |
| 5840 | gccctagggtttccatagacc | 21 | 62.9 | 57.1 | 637/91 | 69 | FAM20C:NM_020223:exon6:c.1118delC:p.T373Rfs*57 | 295460-296260 chromosome 7, GRCh37.p13 Primary Assembly |
| 5841 | agtaaggacgccctctgc | 18 | 62.3 | 61.1 |  |  |  |  |
| 5842 | cgaagttccggaggctgat | 19 | 62.7 | 57.9 | 652/91 | 68 | BEST4:NM_153274:exon8:c.G1058A:p.W353X | 45249991-45250791 chromosome 1, GRCh37.p13 Primary Assembly |
| 5843 | cctataggtggctgaacagatca | 23 | 61.9 | 47.8 |  |  |  |  |
| 5844 | tgtactccgccagttgcttt | 20 | 62.6 | 50.0 | 175/84 | 67 | SHF:NM_138356:exon2:c.G137A:p.W46X | 45490986-45491286 chromosome 15, GRCh37.p13 Primary Assembly |
| 5845 | gcataggaaacgggcatcc | 19 | 61.8 | 57.9 |  |  |  |  |
| 5846 | cagtactcggctaaccctctt | 21 | 61.8 | 52.4 | 739/89 | 68 | MMP9:NM_004994:exon5:c.T758A:p.L253X | 44639490-44640290 chromosome 20, GRCh37.p13 Primary Assembly |
| 5847 | actggcagggtttcccat | 18 | 62.1 | 55.6 |  |  |  |  |

**PCR amplification result**


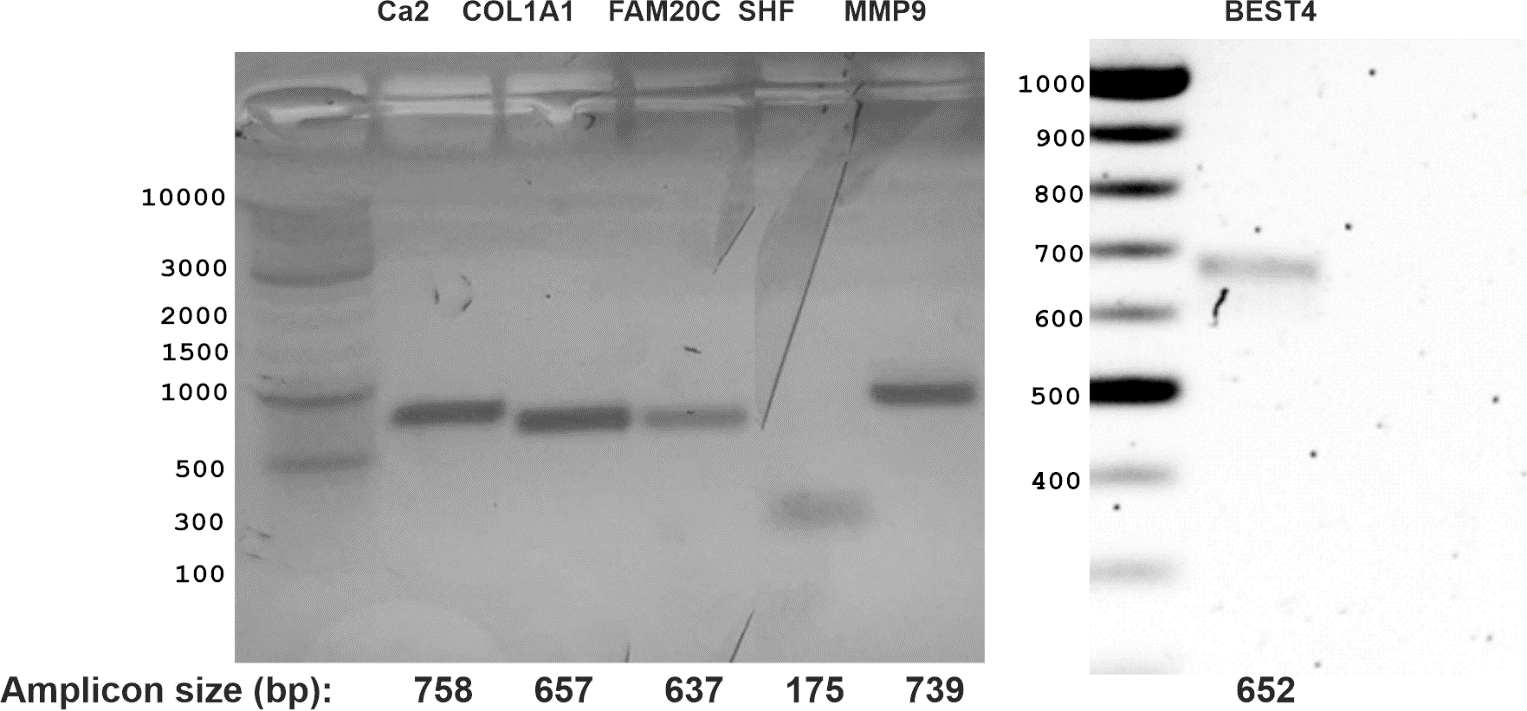


PCR primers combinations:

1. 5836-5837 CA2 (758bp);
2. 5838-5839 COL1A1 (657 bp);
3. 5840-5841 FAM20C (637 bp);
4. 5844-5845 SHF (175 bp);
5. 5846-5847 MMP9 (739 bp);
6. 5842-5843 BEST4 (652 bp) - work with 5xGC buffer;

**PCR:** 210 µl reaction mix contain: **5 µl DNA** (human sample), 73 µl MilliQ, 42 µl 5x Phusion HF buffer, 4.2 µl dNTP (10 mM), 2.1 µl Phusion™ High-Fidelity DNA II Polymerase (2 U/µL). Mix, centrifuge briefly and transfer 18 µl mix to PCR tube and add each 6 µl each 1 µM primer (→ 200 nM).

PCR amplification was carried out under the following conditions:

initial denaturation step at 98°C for 60 sec, followed by 32 amplifications at 98°C for 5 s, at **60** °C for 30 s, and at 72°C for 10 s, followed by a final extension of 72°C for 1 min.

**5842-5843 BEST4 (652 bp) - work with 5xGC buffer:** initial denaturation step at 98°C for 60 sec, followed by 33 amplifications at 98°C for 10 s, at **64** °C for 30 s, and at 72°C for 10 s, followed by a final extension of 72°C for 1 min.

**Results obtained by *in silico* PCR**

**Config.file content**:

targets_path=E:\Genomes\GRCh38.p14\

primers_path=G:\Shared drives\InSilicoPCR\Result\Human\primers.txt

type=primer/probe

linkedsearch=false/true

molecular=linear/circle

number3errors=1

minlen=100

maxlen=1000

FRpairs=false/true

ShowPrimerAlignment=true/false

ShowOnlyAmplicons=true/false

ShowPCRProducts=true/false

ShowPrimerAlignment=true/false

ShowPrimerAlignmentPCRproduct=true/false

ShowPCRproductCalculation=true/false

**Command line:**

java -jar C:\MyPrograms\Java\virtualPCR\dist\virtualPCR.jar C:\MyPrograms\Java\virtualPCR\task\config.file

PCR primers:

**1. 5836-5837 CA2 (758 bp)**

*In silico* Primer(s) search for: ////NC_000008.11 Homo sapiens chromosome 8, GRCh38.p14 Primary Assembly:

>85473967-85474724 Amplicon size: 758bp Ta=60°C

gtgacatcatacttaacgctgcaaaactttctctactgtctccaactccaccatttgcaaaaagtaaacggactcaaactggaggatcctgttttaacagaaatttagaaataaaatgtgtgccttctgtcaaaactgtacatttatttgtcttagagttattgatgaaaacacttgctgttataccaagtactgtgtggatgattagaattaaaatgaaaggaaaattttgatttaaaattatccatattttcaatttcctgagtaacctttattgtgagaaaaagaccattgaataaaatctgtcagctttgattatgtaaatcactcactgtggctttgtctcttcggccttagcttcacttggttcactggaacaccaaatatggggattttgggaaagctgtgcagcaacctgatggactggccgttctaggtatttttttgaaggttagttgatgacccaattcttttttttccctatttttaataaagaatgaccagacagagtatttgtaacatacaggacattctacaaaagagcttaggaaatgcctttgtccctgaaatgttttcaagtttatctcctcctttcatatctgctagttgcagtggagatggagggcagaaagacaataggagaggtacttatttggtaccttttggatgtaaatgtgaagaacataaagagatcaacttgggtgactgctaggtgtttgtttgttttcttttcatttaacctgaggctagacagtac

The number of amplicons = 1

758bp Ta=60°C

Time taken: 5 seconds

**2. 5838-5839 COL1A1 (657 bp)**

__________________________________________________

*In silico* Primer(s) search for: ////NC_000017.11 Homo sapiens chromosome 17, GRCh38.p14 Primary Assembly:

>50186438-50187094 Amplicon size: 657bp Ta=61°C

gtctccatgttgcagaagactttgatggcatccaggttgcagccttggttggggtcaatccagtactctcctgtggtagggcagggcaagatggagtcagggaaagggagcagccagcaccatatggtaggggcacatatgggcatggggaccctggcatggcaggagtaggagggagggagaggctagggcaggccctcaccactcttccagtcagagtggcacatcttgaggtcacggcaggtgcgggcggggttcttgcggctgccctctgggctccggatgttctcgatctgctggctcaggctcttgagggtggtgtccacctcgaggtcacggtcacgaaccacattggcatcatcagcccggtagtagcggccaccatcgtgagccttctcttgaggtggctggggcaggaagctgaagtcgaaaccagcgctgggaggaccagggggaccaggaggtccaggagggccggggggaccctgcacagagagggaagagagtggggattaccggcatccaagtgctttgggggctggagggccatgagcagaggggatgaggggctacatacaacaggaccagcatcaccagtgcgaccgcgaggaccagggggcccaatggggccagggagaccgttgagtccatctttg

The number of amplicons = 1

657bp Ta=61°C

Time taken: 2 seconds

**3. 5840-5841 FAM20C (637 bp)**

__________________________________________________

*In silico* Primer(s) search for: ////NC_000007.14 Homo sapiens chromosome 7, GRCh38.p14 Primary Assembly:

>255575-256212 Amplicon size: 638bp Ta=63°C

gccctagggtttccatagaccagcccctgattttcgagggttcaggccgggctctgcctctggggagctgtgtggcctcgggacgtcagctctgtgggttcatttttctgtaccgtggggtggaggtgtttgctgggattgatggggaactgtgggtgagtcctgcccatgagaagcaccaggcagagcccggcccggccttgggggccgtgagaccacaggtgaggacgcagccctgtgcggccctggtaacccgcagcctgtccctccccagccaacaacatctgcttctacggcgagtgttcctactactgctccacggagcacgccctgtgcgggaagccagaccagatcgagggctcgctggcggccttcctgcccgacctgtccctggccaagaggaagacctggcggaacccttggcggcgttcctaccacaagcgcaagaaggccgagtgagtgcggggccggggggctggcgtccggccaccctacggcagagggagctgggcctgggcgggcatgggagggtcggcgcccacgggggtggcagagatgggtgcagagcctgctgtgcgatgctggcctgtgtgagatgaccgcttcctgatgagacggtggcagagggcgtccttac

The number of amplicons = 1

638bp Ta=63°C

Time taken: 5 seconds

**4. 5842-5843 BEST4 (652 bp) - work with 5xGC buffer**

__________________________________________________

*In silico* Primer(s) search for: ////NC_000001.11 Homo sapiens chromosome 1, GRCh38.p14 Primary Assembly:

>44784341-44784992 Amplicon size: 652bp Ta=61°C

cgaagttccggaggctgatggccggggagggcgcccctacgcccaggaagcggccgagcaacggggtctgcgcggcgggcgcgggccgaccagatccgggggacgcctccacctgcaggctctgctcagggtcgtcgctcatgctgcgggcgggagggcgggctgagccggggcacagggcgggagcggggacgcgggatcggctctcggggaaggaccgaggcatcggtgccccagaggctgagcgaggacccgcgtgccgggtcaggcccagtgcaagccactcaccgcaggttgaaggtggagcccaggaatgagggccgcagagactcggccgccgtggccacagtgtagggtggctgcggctggtcctcatcccagtactggtccttctcagcggggggaaggttctggtacatttcgtccacggatagcagggacacctgggccaccagcaagttacaaggatcctcctctcctctccttcccacggccgggctgagagctgaccgggagagggggcccaggagctgccctggactcctgatcctgatgcttgctcacctgcaagttgcggtctatgagctgatttgtctcaaagtcgtcatcatcctcaccaaatgggttgatgatctgttcagccacctatag

The number of amplicons = 1

652bp Ta=61°C

Time taken: 9 seconds

**5. 5844-5845 SHF (175 bp)**

__________________________________________________

*In silico* Primer(s) search for: ////NC_000015.10 Homo sapiens chromosome 15, GRCh38.p14 Primary Assembly:

>45198820-45198994 Amplicon size: 175bp Ta=61°C

tgtactccgccagttgcttttcttcttctgttttggcccattagccacgggctgtggtgatgatgagatgggctcccggtgagcgcagtgggagtttaggggagacggcgtgagcatccaggaatggggctgggcggaactcagactacccttgggggatgcccgtttcctatg

The number of amplicons = 1

175bp Ta=61°C

Time taken: 3 seconds

**6. 5846-5847 MMP9 (739 bp)**

__________________________________________________

*In silico* Primer(s) search for: ////NC_000020.11 Homo sapiens chromosome 20, GRCh38.p14 Primary Assembly:

>46010880-46011618 Amplicon size: 739bp Ta=62°C

cagtactcggctaaccctcttcctctcgacctgtttcttcagagcacggagacgggtatcccttcgacgggaaggacgggctcctggcacacgcctttcctcctggccccggcattcagggagacgcccatttcgacgatgacgagttgtggtccctgggcaagggcgtcggtgagattctgagtcctcctggcccctgattcccttcattctctcccactcatcacccgccgccctaactccggtcccccctcctcctgcagtggttccaactcggtttggaaacgcagatggcgcggcctgccacttccccttcatcttcgagggccgctcctactctgcctgcaccaccgacggtcgctccgacggcttgccctggtgcagtaccacggccaactacgacaccgacgaccggtttggcttctgccccagcgagagtgagtgagggggctcgccgagggctgggggcgcccaccacccttgatggtcctgggttctaattccagctctgccactagtgctgtgtggcctgcaattcaccctcccgcactctgggcccaattttctcatctgagaaatgatgagagatgggatgaactgcagaccatccatgggtcaaagaacaggacacacttgggggttataatgtgctgtctccgccttctccccctttcccacatcctcctcgccccaggactctacacccaggacggcaatgctgatgggaaaccctgccag

The number of amplicons = 1

739bp Ta=62°C

Time taken: 2 seconds
